# TRPV4 and TRPM8 as putative targets for chronic low back pain alleviation

**DOI:** 10.1007/s00424-020-02460-8

**Published:** 2020-09-21

**Authors:** Stefania Fozzato, Nicolò Baranzini, Elena Bossi, Raffaella Cinquetti, Annalisa Grimaldi, Paola Campomenosi, Michele Francesco Surace

**Affiliations:** 1 Department of Medicine and Surgery, University of Insubria, Varese, Italy; 2 Department of Biotechnology and Life Sciences, University of Insubria, Via Dunant 3, 21100 Varese, VA Italy; 3 Center for Neuroscience Research, University of Insubria, Via Dunant 3, 21100 Varese, VA Italy; 4 Interdisciplinary Research Centre for Pathology and Surgery of the Musculoskeletal System, University of Insubria, Varese, Italy

**Keywords:** Transient receptor potential, Chronic low back pain, TRPV4, TRPM8, Expression, Targets

## Abstract

The purpose of this study is to investigate the presence of nervous fibers and expression of TRP channels in samples harvested during decompressive/fusion spine surgeries from patients affected by chronic low back pain (CLBP). The aim was to understand if members of this family of receptors played a role in detection and processing of painful stimuli, to eventually define them as potential targets for CLBP alleviation. Expression of transient receptor potential (TRP) channels (A1, V1, V2, V4, and M8) was evaluated in samples from different periarticular sites of 6 patients affected by CLBP, at both protein and transcript levels. The capsular connective pathological tissue appeared infiltrated by sensitive unmyelinated nervous fibers. An increase in TRP channel mRNAs and proteins was observed in the pathological capsule compared with tissues collected from the non-symptomatic area in five of the six analyzed patients, independently by the location and number of affected sites. In particular, TRPV4 and TRPM8 were consistently upregulated in pathological tissues. Interestingly, the only patient showing a different pattern of expression also had a different clinical history. TRPV4 and TRPM8 channels may play a role in CLBP and warrant further investigations as possible therapeutic targets.

**Electronic supplementary material:**

The online version of this article (10.1007/s00424-020-02460-8) contains supplementary material, which is available to authorized users.

## Introduction

Chronic low back pain (CLBP) is a painful condition arising from spinal structures such as bones, joints, muscles, tendons, ligaments, and intervertebral disks due to traumatic, degenerative, or inflammatory diseases. CLBP is a highly prevalent condition associated with disability, work absenteeism, and huge healthcare costs [36].

Its onset and regulatory mechanisms are not properly understood, due to the multiple factors concurring in its pathogenesis, such as the neuroinflammatory peripheral pathways [1].

The inflammatory response prompts the release of an array of molecules that acts in altering the expression and modulating the function of various ion channels as the transient receptor potential (TRP) ion channels, sodium channels, and mechanosensitive ion channels in nociceptors inducing sensitization, pain hypersensitivity, or hyperalgesia [7, 35].

Under pathophysiological conditions, TRP channels are sensitized, their activation threshold reduced, and, consequently, perception of painful (hyperalgesia) and non-painful (allodynia) stimuli enhanced [19, 21, 25].

The peripheral sensitization in primary sensory neurons together with central sensitization induces neuronal plasticity in pain-coding pathways. Plasticity is commonly considered a participant in chronic pain onset [3, 12].

To understand the players involved in peripheral pain hypersensitivity, the altered expression of selected membrane receptors was investigated in specimens from patients of different ages and genders, with confirmed diagnosis of CLBP. TRPV1–4, TRPA1, and TRPM8 were selected because of their expression in peripheral sensory neurons as molecular nociceptors, actively transducing thermal, chemical, and mechanical stimuli [13, 24], thus being involved in CLBP. For example, TRPV4 acts as a sensor of mechanical or osmotic signals and it is present not only in the nervous system but also in several musculoskeletal tissues, including cartilage, bone, and synovium. This protein was shown to have altered expression in pathological conditions and to have a role in pain perception in CLBP [26].

Also, TRPV1 is known to be stimulated by several inflammatory neuropeptides and signaling molecules [14] and overexpressed in osteoarthritis [38, 39]; thus, it is reasonable to expect a similar pattern of expression in specimens retrieved from patients affected by CLBP. Moreover, in inflammatory tissue, it is often co-expressed with TRPA1 [5] that can be equally stimulated by an array of molecules present in inflammation and in acute mechanical hypersensitivity [2, 13, 41]. Finally, many papers reported that also TRPM8 is expressed on both Aδ and C fiber and overexpressed in pain conditions, where it plays a role in amplifying pain sensation after injury, particularly in models of neuropathic pain. To date, the role of TRPM8 is not completely understood: some data show that TRPM8 is active in reducing pain; others suggest that TRPM8 increases pain after injury [4, 11, 24, 42].

In order to shed light on the role played by different TRP channels in the detection and processing of painful stimuli, and possibly to identify novel therapeutic targets, we investigated their expression in samples from patients affected by CLBP surgically treated. The surgical technique for the placement of bilateral pedicle screws implies their positioning in the adjacent vertebrae. Therefore, there is the opportunity to collect pathological tissue samples from the symptomatic site but also from some non-symptomatic contralateral or adjacent level, these tissues that were used as controls from the same patient. Harvested samples were evaluated for morphological, ultrastructural, histochemical, and immunohistochemical alterations. The expression of several members of the TRP family ion channels was evaluated for immunohistochemical and gene expression analyses.

## Materials and methods

**Fig. 1:**
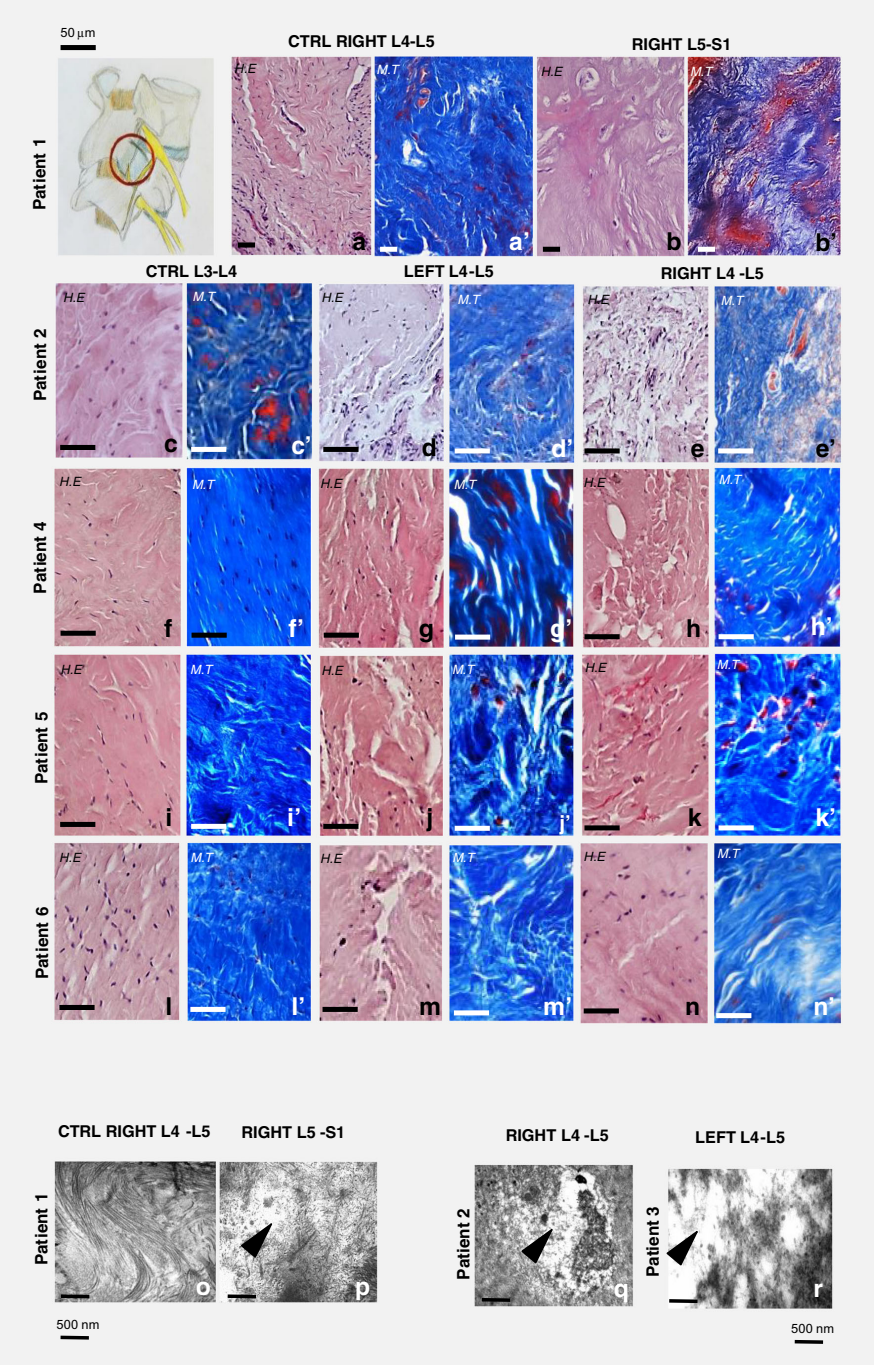
Morphological, colorimetric, and ultrastructural analyses of control and pathological joint connective tissues. In the top-left drawing, the site from which tissue samples were harvested is shown (encircled). H.E. (staining connective tissue in pink and cells nuclei in violet), M.T. (staining collagen in blue and muscle in red) (**a**, **a′**; **c**, **c′**; **f**, **f′**; **i**, **i′**; **l**, **l′**), and TEM ultrastructural analysis (**o**) of control connective tissues showed compact and well-organized collagen. A loose and disorganized connective tissue was instead visible in H.E.- and M.T.-stained pathological tissues (**b**, **b′**; **d**, **d′**; **e**, **e′**; **g**, **g′**; **h**, **h′**; **j**, **j′**; **k**, **k′**; **m**, **m′**; **n-n′**). Ultrastructural analysis at TEM (**p**, **q**, **r**) of pathological sections in different patients highlighted that collagen fibers lost the characteristic spatial organization (arrowhead in **p**, **q**, **r**). Bars in **a**–**n**′: 50 μm; bars in **o**–**r**: 500 nm

Samples of connective tissues were retrieved from six patients affected by CLBP, caused by degenerative disk disease, segmental instability, interapophyseal arthritis, and degenerative lumbar pathology. Fragments of periarticular tissue were collected from joints during the necessary surgical decompression. Harvesting site is shown in Fig. 1. The surgical technique for the placement of bilateral pedicle screws requires “reshaping” the adjacent interapophyseal joints, contralateral or cranial level, in order to free the access to pedicles. Therefore, it is possible to collect pathological tissue samples from the symptomatic sites but also from the non-symptomatic ones. The samples harvested from the latter were used as controls, without any damage for the patient.

The samples available were dependent on the amount of material removed during the surgical procedure without any damage for the patient.

The dissected joint tissues were processed for histological, immunohistochemical, ultrastructural, and gene expression analyses. The samples obtained from patients 1 and 3 were insufficient for a complete analysis, and only immunohistochemical and gene expression analysis were respectively performed in these cases. All patients were adequately informed of the study and signed an informed consent to donate samples for the present research. Clinical data of patients included in the study are reported in Supplementary File 1.

### Histological and histochemical analyses at light microscopy

For histological analysis at light microscopy, samples were fixed with 4% paraformaldehyde overnight at 4 °C, embedded in paraffin, and serial-sectioned (5 μm) with a Leica Jung Multicut 2045 Microtome (Leica, Wien, Austria). After deparaffinization and rehydration through a graded ethanol scale, sections were stained with hematoxylin and eosin (H.E.) (Bio-Optica, Milan, Italy), for a general morphological view, with Masson Trichrome (M.T.) with aniline blue kit (Bio-Optica, Milan, Italy) to highlight the collagenic component of capsular connective tissue and silver impregnation (S.I.) histoenzymatic kit (Bio-Optica, Milan, Italy) to highlight argyrophilic neurofibrils in the capsular connective. Samples were observed with a Nikon Eclipse Ni light microscope (Nikon, Tokyo, Japan) and data were recorded with a DS-5M-L1 digital camera system (Nikon, Tokyo, Japan).

### Ultrastructural analysis at transmission electron microscopy (TEM)

For ultrastructural analysis, samples were fixed for 2 h in 0.1 M cacodylate buffer at pH 7.4, containing 2% glutaraldehyde. Specimens were then washed in the same buffer and post-fixed for 1 h with 1% osmium tetroxide in cacodylate buffer, pH 7.4. After standard ethanol dehydration, specimens were embedded in an Epon-Araldite 812 mixture. Ultrathin sections (80 nm) were obtained with a Reichert UltraCut S ultramicrotome (Leica, Nussloch, Germany), stained by uranyl acetate and lead citrate, and observed with a JEOL JEM-1010 EX transmission electron microscope (JEOL USA, Inc., Peabody, MA, USA). Data were recorded with a MORADA digital camera system (Olympus, Tokyo, Japan).

### Immunofluorescence analysis

For immunofluorescence analysis, samples were fixed with 4% paraformaldehyde for 1 h [28], embedded in paraffin, and serial-sectioned (5 μm) with a Leica Jung Multicut 2045 Microtome (Leica). After deparaffinization and rehydration through a graded ethanol scale, sections were immersed in 10 mM sodium citrate buffer (pH 6.0) for 10 min in a microwave oven for antigen retrieval and then incubated for 30 min with a blocking solution (2% bovine serum albumin (BSA) and 0.1% Tween20 in phosphate-buffered saline (PBS), 138 mM NaCl, 2.7 mM KCl, 4.3 mM Na_2_HPO_4_, 1.5 mM KH_2_PO_4_, pH 7.4). Sections were then incubated for 1 h at 37 °C [44] with primary antibodies (Alomone Labs, Jerusalem, Israel) all diluted 1:200 in blocking solution. The primary antibodies used are presented in Supplementary Table 1.

After washing with PBS, the specimens were incubated for 1 h at room temperature with goat anti-rabbit Cy3-conjugated antibodies (Abcam, Cambridge, UK, excitation 562 nm, emission 576 nm), diluted 1:250 in blocking solution. Nuclei were stained by incubating for 15 min with 49.6-diamidino-2-phenylindole (DAPI) 100 ng/ml in PBS (Sigma-Aldrich, Milan, Italy). Slides were mounted with CitiFluor (CitiFluor Ltd., UK) and examined with the Nikon Eclipse Ni fluorescence microscope (Nikon, Tokyo, Japan).

### RNA extraction and qPCR analysis

Tissues for RNA extraction were weighted and grinded in liquid nitrogen using mortar and pestle. One milliliter of TRI reagent (Sigma-Aldrich, Milan, Italy) was added for every 100 mg starting tissue and RNA was extracted following manufacturer instructions. RNA samples were quantified with a Quantus Fluorometer (Promega, Milan, Italy) and run on an agarose gel for quality control. For real-time quantitative PCR (qPCR), cDNA was obtained from 750 ng of RNA by using the iScript gDNA Clear cDNA synthesis kit (Bio-Rad, Milan, Italy). Gene expression analysis was performed in triplicate using a CFX96 thermal cycler (Bio-Rad, Milan, Italy) and the iTaq Universal SYBR Green Supermix (Bio-Rad, Milan, Italy). Primers for the genes under investigation were designed to have at least one of the primers in the pair designed on an exon-exon junction, or to encompass at least one intron. For primer design and thermodynamic analysis of their quality, the following programs were used: the Primer-Blast tool at NCBI (http://www.ncbi.nlm.nih.gov/tools/primer-blast/), the OligoCalc (http://biotools.nubic.northwestern.edu/OligoCalc.html), and the IDT SciTools (http://eu.idtdna.com/pages/scitools). Primer sequences are reported in Supplementary Table 2.

Relative mRNA quantification was obtained by applying the 2^-DDCq method [23].

Suitability of this normalization method was investigated by (i) evaluating the stability of candidate reference genes across samples, including both pathological and control tissues by the GeNorm algorithm (Supplementary Fig. 2). *GAPDH* was excluded, whereas *B2M* and *HPRT1* were both confirmed as suitable reference genes. It was also investigated by (ii) checking the efficiency of the assays by constructing calibration curves (Supplementary Fig. 3) and calculating efficiency as (10^–1/slope^ − 1) * 100. Efficiency was comprised between 95 and 105% for all assays. Of the two reference genes, *B2M* had the efficiency most similar to those of the genes of interest; hence, *B2M* was subsequently used for data normalization. Melting curve analysis was performed to ensure that single amplicons were obtained for each target (Supplementary Fig. 4).

### Quantification of silver impregnation, immunofluorescence reaction, and qPCR

Black staining and fluorescence intensity were assessed using the ImageJ software package (http://rsbweb.nih.gov/ij/download.html), in order to standardize quantification. The percentage of nerve fibers and of fluorescence intensity were assessed by analyzing 5 random fields of 45.000 μm^2^ for each slide. Graphical representation of quantified molecules was performed with GraphPad Prism version 8.4.2.

## Results

### Morphological analysis of capsular connective tissue

Hematoxylin and eosin (Fig. 1a–n) and Masson Trichrome (Fig. 1a′–n′) stains were performed to evaluate any gross morphological changes in the joint tissues deriving from the pain-affected sites compared with control ones.

In all five analyzed patients, the control joint level appeared to be composed of compact and well-organized connective tissue, in which numerous cell nuclei were distinguishable (Fig. 1a, c, f, i, l). As highlighted by the aniline blue component of Masson’s trichrome staining, the main constituent of joint tissue was collagen that appeared organized in compact bundles (Fig. 1a′, c′, f′, i′, l′). Conversely, the connective tissue samples from pain-affected areas appeared more disorganized. As shown in H.E.-stained (Fig. 1b, d, e, g, h, j, k, m, n) and M.T.-stained (Fig. 1b′, d′, e′, g′, h′, j′, k′, m′, n′) tissues, numerous empty spaces were visible between the bundles of collagen fibers. Ultrastructural analysis at TEM confirmed the compact and well-organized collagenic composition of the control connective tissue (Fig. 1o). Pathological tissues from different patients presented some areas where collagen fibers were bundled and spatially organized and other areas with connective tissue rarefaction, fragmented and disorganized collagen fibers, when analyzed by TEM (Fig. 1p, q, r).

**Fig. 2:**
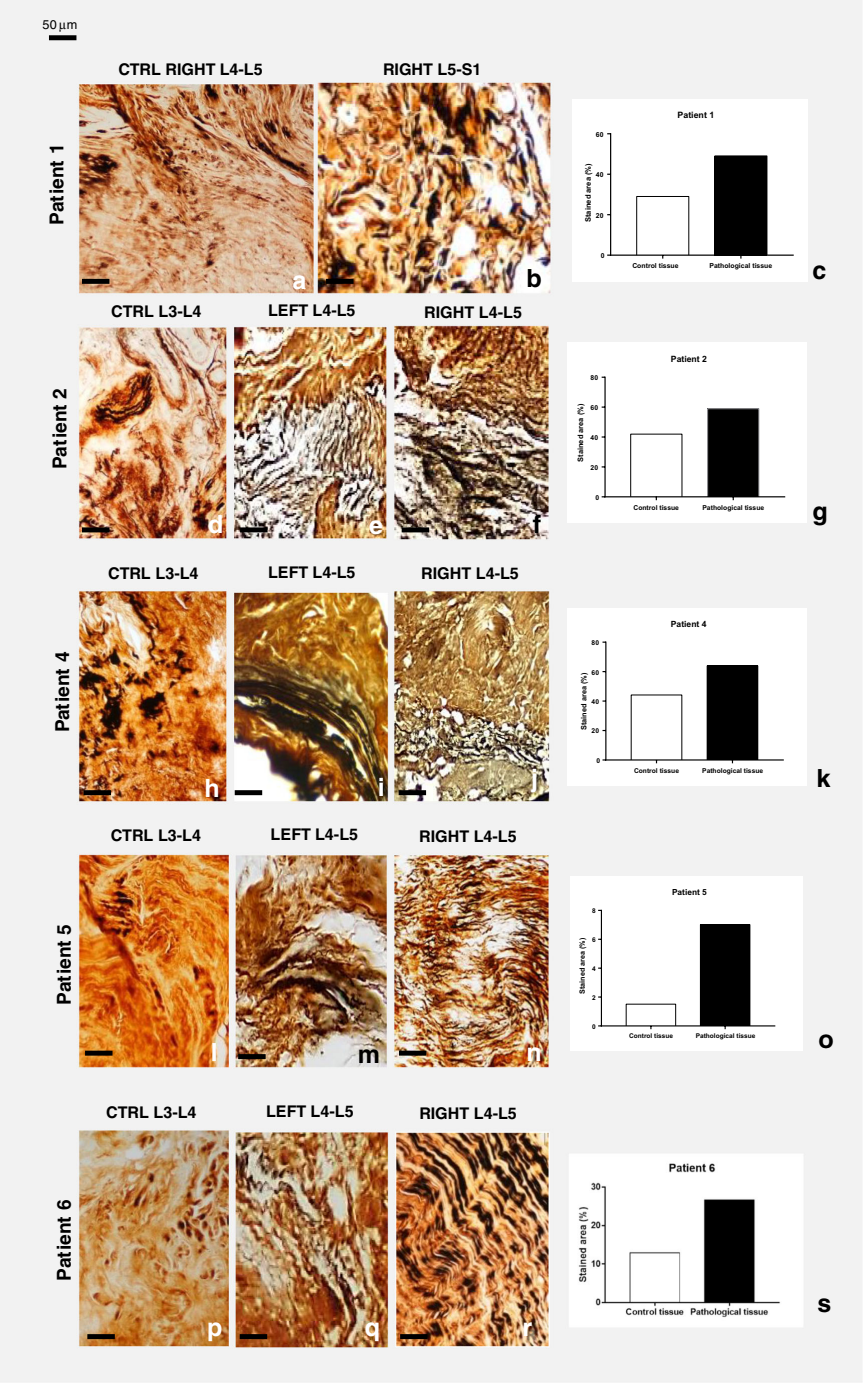
Silver impregnation staining of control and pathological joint connective tissue. Compact and well-organized collagen (in light brown) and few black staining for nervous fibers were observed in control connective tissue (**a**, **d**, **h**, **l**, **p**). Numerous black nerve fibers were instead visible in the loose and disorganized pathological connective tissue (**b**, **e**, **f**, **i**, **j**, **m**, **n**, **q**, **r**). Bars in stained tissues: 50 μm. The graphs (**c**, **g**, **k**, **o**, **s**) show the amount of black staining significantly increased in affected joint samples compared with control tissues, measured by ImageJ software package

Silver impregnation (S. I.), which selectively distinguishes connective tissue (brown colored) from argent affine nervous fibers (black colored), detected only a few fibers in control samples (Fig. 2a, d, h, l, p). Conversely, affected tissues were infiltrated by numerous nerve fibers (Fig. 2b, e, f, i, j, m, n, q, r). Furthermore, the amount of black staining, quantified by analyzing 5 random fields of 45.000 μm^2^ for each slide using ImageJ software package, significantly increased in pain-affected joint samples compared with that in control tissue and the differences were graphically represented (Fig. 2c, g, k, o, s). This finding seemingly supported the hypothesis of a greater infiltration of sensory nerves in the pathological joint tissue.

### Immunofluorescence and mRNA expression analyses

In order to correlate the possible increase of nerve fiber infiltration with an increased pain sensation, we performed immunolocalization of TRPA1, TRPV1, TRPV2, TRPV4, and TRPM8 on control and affected tissues of each patient. In addition, transcripts levels of TRP channels (A1, V1, V2, V4, and M8) in almost all tissues deriving from the pain-affected regions were assessed by qPCR*.*

**Fig. 3:**
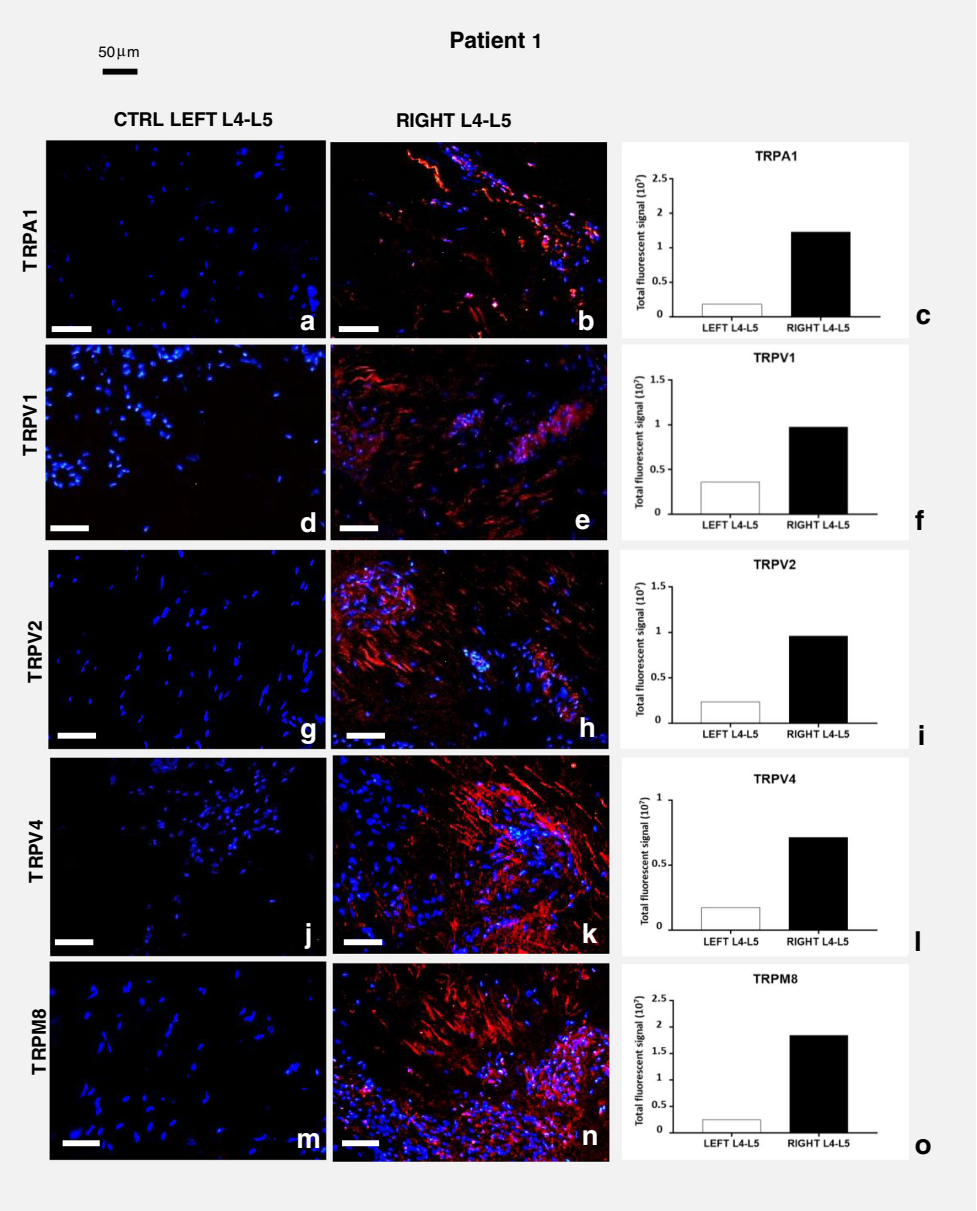
Expression of TRP channel proteins in patient 1. Immunolocalization in control (**a**, **d**, **g**, **j**, **m**) and pathological (**b**, **e**, **h**, **k**, **n**) joint tissues from patient 1. Receptors stained in red, nuclei counterstained in blue (DAPI). Bars: 50 μm. The graphs (**c**, **f**, **i**, **l**, **o**) show the amount of immunofluorescence in control and pathological tissues for each TRPs analyzed, measured by ImageJ software package, increased in affected joint samples compared with control tissues. See text for details

In patient 1 (Fig. 3), all samples deriving from pathological tissue (Fig. 3b, e, h, k, n) showed higher expression of all tested TRP receptors, compared with control tissue (Fig. 3a, d, g, j, m). As very small samples were retrieved from this patient, no molecular analysis was performed. The quantification of immunofluorescence signal of each receptor in pathological and control tissue was measured by ImageJ software package and graphically represented (Fig. 3c, f, i, l, o).

**Fig. 4:**
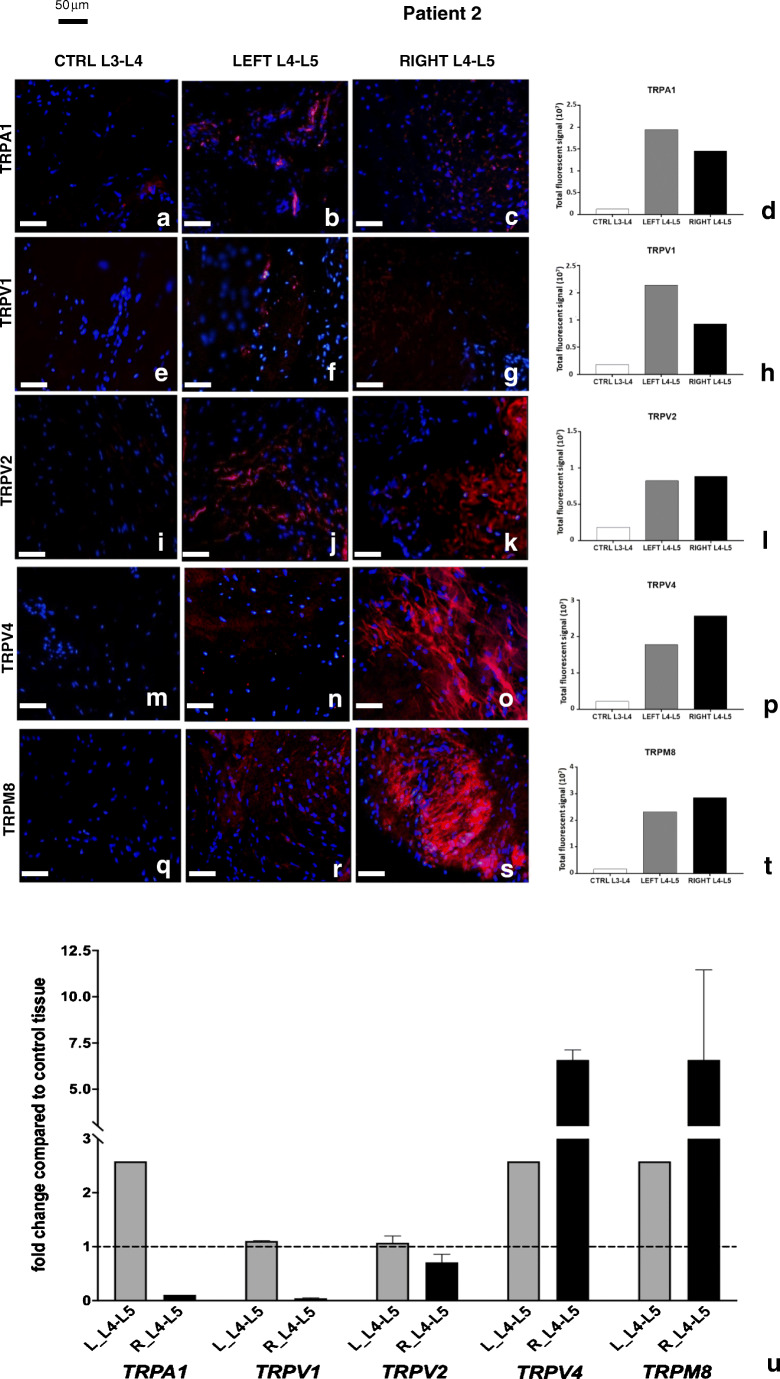
Expression of TRP channel proteins and transcripts in patient 2. Immunolocalization of TRPs in control (**a**, **e**, **i**, **m**, **q**) and pathological (**b**, **c**, **f**, **g**, **j**, **k**, **n**, **o**, **r**, **s**) joint tissues from patient 2. Receptors stained in red, nuclei counterstained in blue (DAPI). Bars: 50 μm. The graphs (**d**, **h**, **l**, **p**, **t**) show the amount of immunofluorescence in control and pathological tissues for each TRPs analyzed, measured by ImageJ software package, increased in affected joint samples compared with control tissues. Expression of TRP channels’ transcripts in different samples from patient 2 (**u**). The fold change in expression in pathological samples compared with control tissue (dotted line) is reported. Left-hand side samples: gray bars; right-hand side samples: black bars. See text for details

In patient 2 (Fig. 4), the immunofluorescence expression of TRPA1 (Fig. 4a, b, c) and TRPV1 (Fig. 4e, f, g) was higher in the left side, while TRPV4 (Fig. 4m, n, o) and TRPM8 (Fig. 4q, r, s) were mainly expressed in the right side, as highlighted by the corresponding graphs (Fig. 4d, h, p, t). In this patient, the immunofluorescence expression level of TRPV2 (Fig. 4i, j, k) was similar in both sides (Fig. 4l).

When transcripts were examined, TRPV4 and TRPM8 were increased in all pathological samples compared with controls, with the highest expression (6.5-fold) on the right side. TRPA1 transcript showed an increase only in the left side affected tissues (Fig. 4u).

**Fig. 5:**
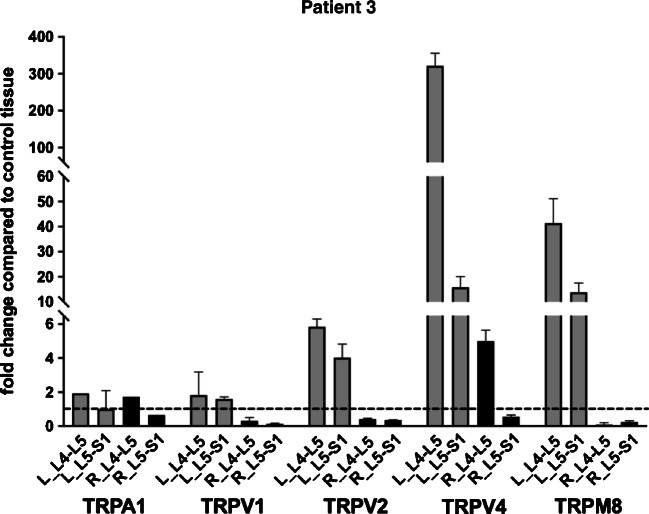
TRP transcripts levels in patient 3. Expression of TRP channels’ transcript in different samples from patient 3. The fold change in expression in pathological samples compared with control tissue (dotted line) is reported. Left-hand side samples: gray bars; right-hand side samples: black bars. See text for details

Samples retrieved from patient 3 were very small and were processed only for gene expression analysis (Fig. 5a). TRPV2, TRPV4, and TRPM8 showed a significant increase in transcript levels in the left side of both affected joint levels (L4-L5 and L5-S1), whereas on the right side, only TRPV4 was increased in the L4-L5 but not L5-S1 level. Overall, the highest increase was observed for TRPV4. Modest changes in expression were observed for TRPA1 and TRPV1.

**Fig. 6:**
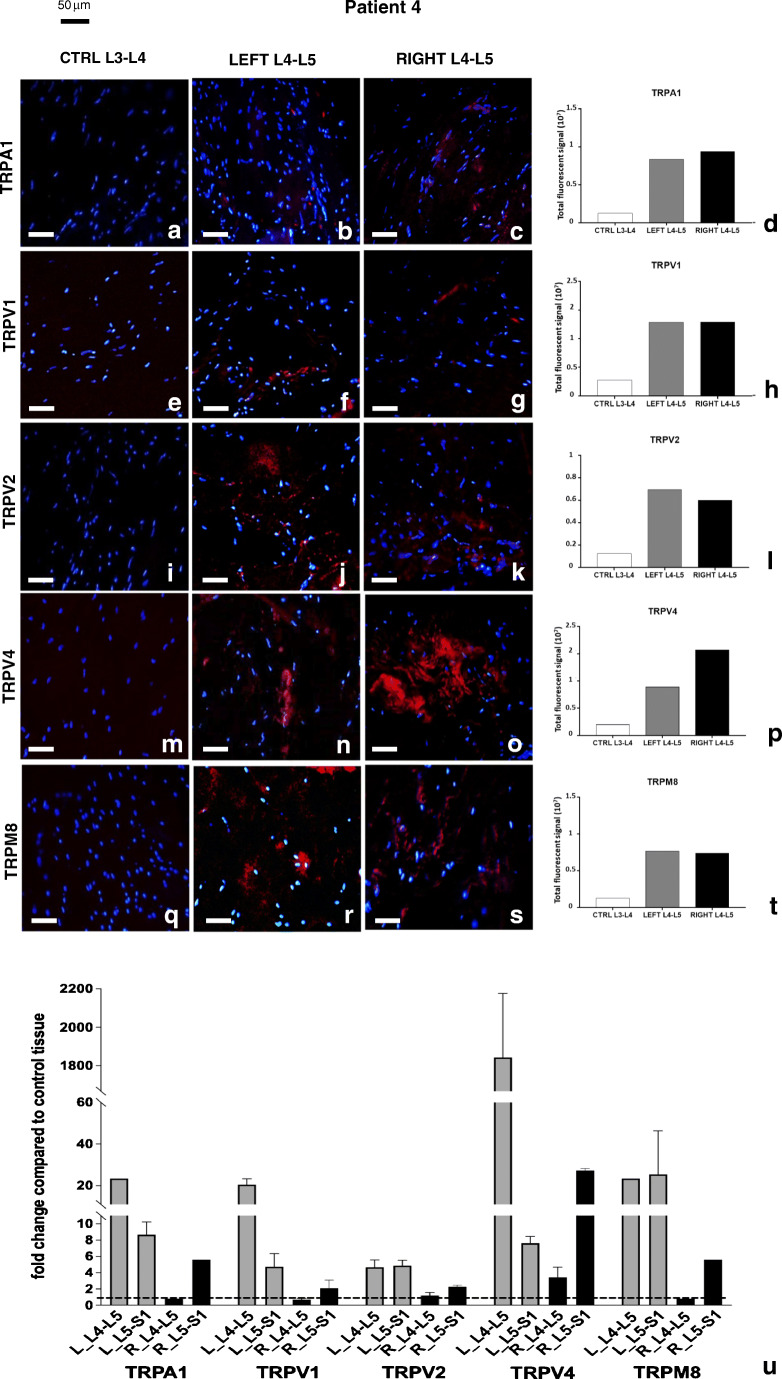
Expression of TRP channel proteins and transcripts in patient 4. Immunolocalization of and TRPs in control (**a**, **e**, **i**, **m**, **q**) and pathological (**b**, **c**, **f**, **g**, **j**, **k**, **n**, **o**, **r**, **s**) joint tissues from patient 4. Receptors stained in red, nuclei counterstained in blue (DAPI). Bars: 50 μm. The graphs (**d**, **h**, **l**, **p**, **t**) show the amount of immunofluorescence in control and pathological tissues for each TRPs analyzed, measured by ImageJ software package, increased in affected joint samples compared with control tissues. Expression of TRP receptors’ transcript in different samples from patient 4 (**u**). The fold change in expression in pathologic samples compared with control tissue (dotted line) is reported. Left-hand side samples: gray bars; right-hand side samples: black bars. See text for details

For patient 4 (Fig.6), an increase in the immunofluorescence of all receptors was observed in the pathological tissues; the increase was particularly evident for TRPV4 (Fig. 6m, n, o), TRPM8 (Fig. 6q, r, s), and TRPV2 (Fig. 6i, j, k). A graphical representation of immunofluorescence quantification for all TRP channels by ImageJ software package is shown in Fig. 6d, h, l, p, t.

The increase in TRPV4 and TRPM8 protein levels correlated with an increase in transcript (Fig. 6u).

**Fig. 7:**
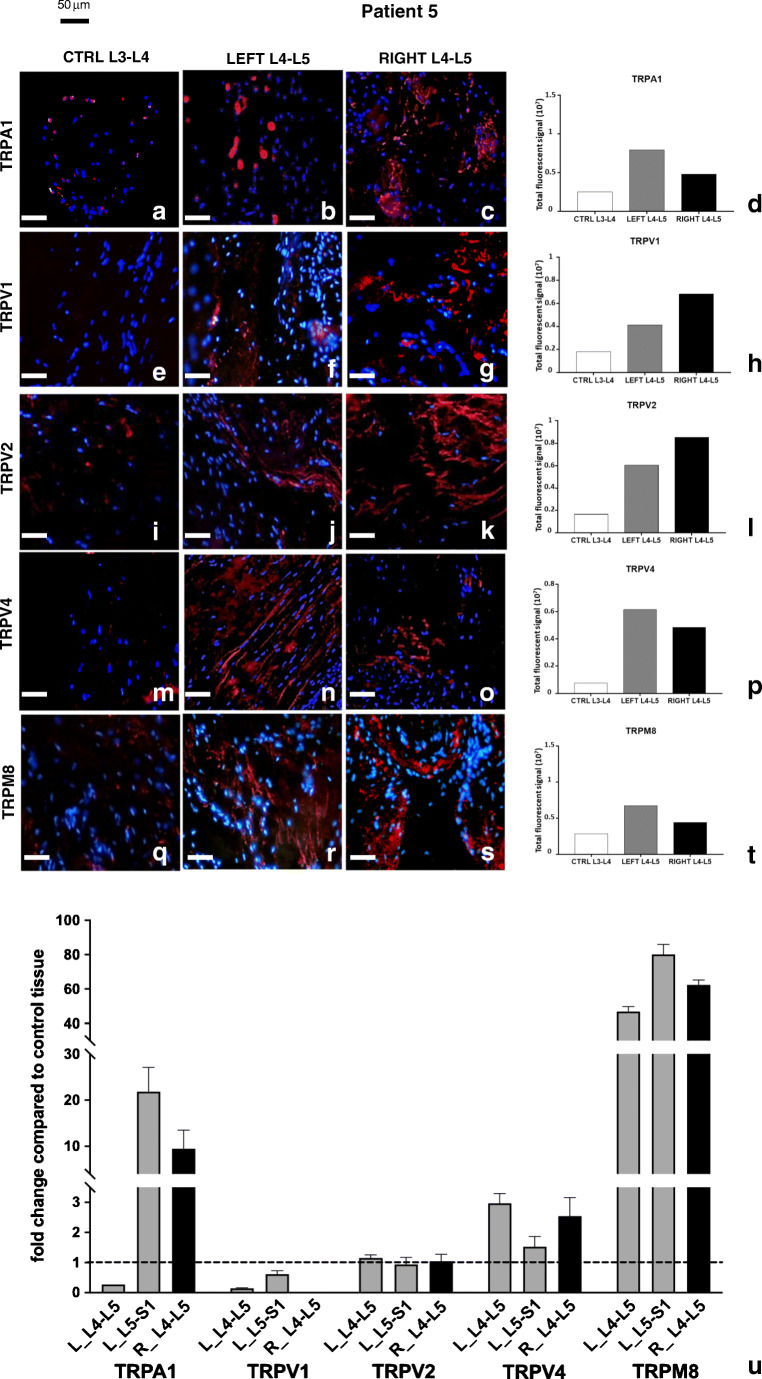
Expression of TRP channel proteins and transcripts in patient 5. Immunolocalization of TRP in control (**a**, **e**, **i**, **m**, **q**) and pathological (**b**, **c**, **f**, **g**, **j**, **k**, **n**, **o**, **r**, **s**) joint tissues from patient 5. Receptors stained in red, nuclei counterstained in blue (DAPI). Bars: 50 μm. The graphs (**d**, **h**, **l**, **p**, **t**) show the amount of immunofluorescence in control and pathological tissues for each TRPs analyzed, measured by ImageJ software package, increased in affected joint samples compared with control tissues. Expression of TRP receptors’ transcript in different samples from patient 5 (**u**). The fold change in expression in pathologic samples compared with control tissue (dotted line) is reported. Left-hand side samples: gray bars; right-hand side samples: black bars. See text for details

In patient 5 (Fig. 7), all pathological samples (Fig. 7b, c, f, g, j, k, n, o, r, s) showed a general increase in all receptors compared with control tissues (Fig. 7a, e, i, m, q). A graphical representation of TRP channel expression is shown (Fig. 7d, h, l, p, t).

At the transcript level, TRPM8 showed the highest and most consistent increase. TRPV4 transcript was increased at the L4-L5 level bilaterally, whereas TRPA1 was less consistently increased. TRPV1 and TRPV2 were unmodified or even decreased (Fig. 7u). TRPA1, TRPV4, and TRPM8 transcript levels correlated with protein levels.

**Fig. 8:**
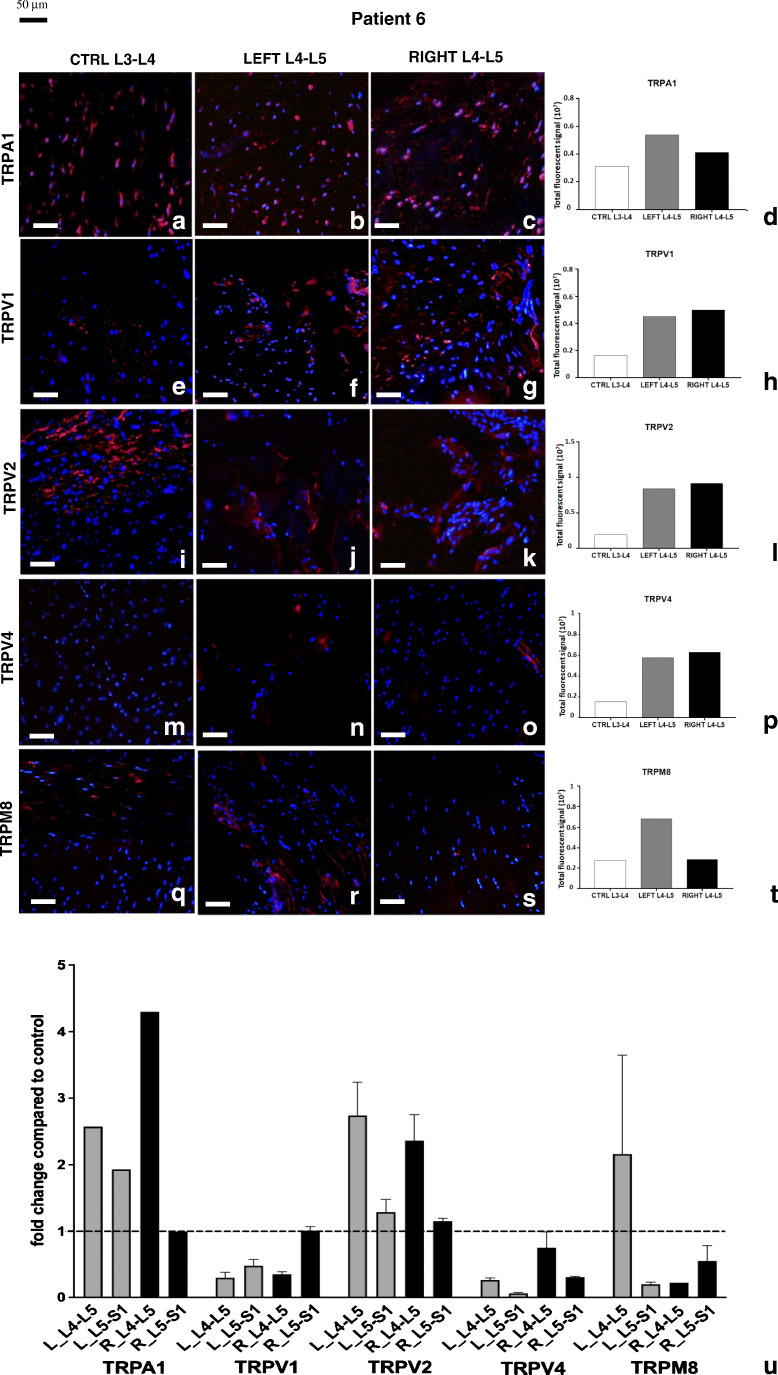
Expression of TRP proteins and transcripts in patient 6. Immunolocalization of TRP in control (**a**, **e**, **i**, **m**, **q**) and pathological (**b**, **c**, **f**, **g**, **j**, **k**, **n**, **o**, **r**, **s**) joint tissues from patient 6. Receptors stained in red, nuclei counterstained in blue (DAPI). Bars: 50 μm. The graphs (**d**, **h**, **l**, **p**, **t**) show the amount of immunofluorescence in control and pathological tissues for each TRPs analyzed, measured by ImageJ software package. Expression of TRP receptors’ transcript in different samples from patient 6 (**u**). Fold change in expression of these receptors in pathologic samples compared with control tissue (dotted line) is reported. Left-hand side samples: gray bars; right-hand side samples: black bars. See text for details

Patient 6 (Fig. 8) showed a different pattern of receptor expression compared with previous patients. TRPA1 (Fig. 8a, b, c) and TRPV1 (Fig. 8e, f, g) expressed levels of proteins similar to other patients (Fig. 8d, h). TRPV4 (Fig. 8m, n, o) and TRPM8 (Fig. 8q, r, s) were almost absent in control tissue and poorly expressed in the pathological samples (Fig. 8p, t). TRPV2 (Fig. 8i, j, k) was almost equally expressed in both sides (Fig. 8l).

At the transcript level, this patient was the only one without increased TRPV4 levels (Fig. 8u). A reduction or no change was observed also for TRPV1 and TRPM8. TRPA1 and TRPV2 showed just a modest increase (less than fivefold) in transcript levels in pathological compared with control tissues and compared with previously described samples.

No signal was detected in negative control experiments where the primary antibodies were omitted, and samples were incubated only with secondary antibodies (Supplementary Fig. 1).

## Discussion

In this work, evidence of morphological alterations of the pathological capsular connective tissue is provided. Indeed, the extracellular matrix appeared degraded in samples from areas affected by chronic pain; significantly greater infiltration of pathological tissues by nervous fibers was observed in all patients. Notably, sprouting of nerve fibers was described as a landmark for articular hypersensitivity and subsequent allodynia [10].

Supporting this hypothesis, the presence of NGF (nerve growth factor) and its receptor TrkA (tyrosine kinase receptor A) in the periarticular and articular tissues of degenerative lumbar facet joints was previously described [35].

Moreover, immunofluorescence analyses highlighted a significant increase in the levels of TRP channels in the pathological tissue. These observations suggest that the TRP channel increase could be related to the higher infiltration by nervous fibers in pathological samples. These results are aligned with previous studies that identified these channels on axons in peripheral tissues [8]. TRP channels act as sensors of various stimuli in peripheral sensory neurons. Their activation and/or sensitization in sensory nerves during inflammation is considered to be the major mechanism underlying neuropathic and inflammatory pain [5, 19, 23, 29].

Increased expression of TRPV4 in pathological samples was the most consistent finding in this study, regardless of the location and number of affected sites in each patient. TRPV4 was previously associated to musculoskeletal diseases, skeletal dysplasia, and arthropathy. Several amino acid substitutions were identified in TRPV4 and related to musculoskeletal disorders [18, 25, 30, 31, 34]. In addition, TRPV4 activation, regulation, and expression vary in accordance with different pathological conditions, such as those provided by the absence of mechanical loading of cartilage [26, 32, 33]. In animal models, TRPV4 was shown to play a role in the response of cartilage to loading, by generating Ca^2+^ transient currents [32]. On the other hand, loss of TRPV4 in adult mice was proven to alleviate age-related, degenerative cartilage changes [33]. The above reported data, together with the results presented here, suggest that TRPV4 could play a major role in joint degenerative changes and the associated inflammation and pain. Conversely, other studies previously stated that diminished TRPV4 function could result in degenerative changes of hyaline cartilage, while hyperactive TRPV4 was associated with alterations of the growth plate cartilage [26]. In any case, targeting TRPV4 function may provide a direct therapeutic approach to treat arthropathies and related pain symptoms.

TRPV4 was shown to be a key component in the transduction of mechanical signals, promoting cartilaginous matrix synthesis in mice [32]. Osteoblasts and osteoclasts express TRPV4 that is involved in the regulation of both bone formation and resorption [27]. In cell culture, elevated TRPV4 mRNA levels were observed during osteoblastic differentiation [37]. In addition, Ca^2+^ signaling and regulation proved to be significant for bone homeostasis and to be affected by mechanical stimuli [20]. Furthermore, calcium waves were induced in osteoblasts expressing TRPV4 when mechanical stimuli were applied. Thus, TRPV4 acts as a mechano-sensor, inducing bone loss under non-loading condition [26].

Interestingly, the increased expression of TRPV4 found in this study in specimens from degenerative joints was also observed in human primary synovial cells isolated from subjects with inflammatory arthropathies [16] this confirms once more that TRPV4 channels may represent an effective target to treat arthropathy [26].

The second receptor most consistently overexpressed in pathological compared with control tissue was TRPM8 that is also known to play a role in detection of mechanical, thermal, and chemical stimuli [5, 22, 40]. Sensor of cold temperature, TRPM8 has voltage-dependent gating properties. TRPM8 is expressed in a subgroup of primary afferent sensory neurons in pathological conditions [9]. In a model of chronic constriction injury, the expression of TRPM8 is increased in sensory neurons [24]. TRPM8 is a good target for treatment of cold allodynia, a frequent aspect of neuropathic pain. Moreover, TRPM8 activity is increased in the presence of nerve growth factor or other neurotrophic factors. TRPM8 has a role in core body temperature regulation and detection of TRM8 antagonist underline its competence in pain-treatment [17, 42]. A pharmacological blockage of TRPM8 signaling determines a reduction in cold hypersensitivity induced by nerve injury [6, 15, 43].

All patients included in this study, except one, showed the same pattern of receptor expression. The exception was represented by patient 6, in whom the expression of TRPV4 and TRPM8 channels was similar to control tissue and TRPA1, TRPV1, and TRPV2 were expressed, although at a lower level, also in the tissue used as control (probably due to an initial degree of osteoarthritis in this tissue). This expression pattern of the receptors compared with the other patients is particularly interesting and could be related to the different clinical histories. More specifically, this patient had previous surgery at the same level with the implant of an interspinous process device which is known to prevent mechanical loading of the posterior joints by blocking extension [36]. This finding suggests that overexpression of TRPV4 and TRPM8 could mainly be due to mechanical stimuli [37, 45]. One of the main limitations of this study is the small number of patients considered and the epidemiological variations such as age, gender, and spinal pathological level. However, the rare occurrence of samples and the availability of control tissues provided a unique opportunity for this pilot study.

## Conclusions

Different types of receptors and of voltage- and ligand-gated ion channels are involved in the detection and processing of painful stimuli at several anatomical sites, such as intervertebral disks, facet joints, and nerve roots.

Our data demonstrate overexpression of TRPs, in particular TRPV4 and TRM8, associated to an increase in nervous fibers, in pathological capsular connective tissues compared with control ones. These results suggest the involvement of TRP channels in sensory nerve function in the context of CLBP, underlining the potential importance of these channels as targets for the next generation of CLBP therapies.

## Electronic supplementary material

ESM 1 (DOCX 1805 kb)
