# Supplementary material for: TRPV4 and TRPM8 as putative targets for chronic low back pain alleviation
Source: Pflugers Arch. 2020 Sep 21;473(2):151–65. doi: 10.1007/s00424-020-02460-8 (PMC7835199; doi:10.1007/s00424-020-02460-8)
Supplement: Supplementary file 1 — (DOCX 1805 kb) [file 424_2020_2460_MOESM1_ESM.docx]

**TRPV4 AND TRPM8 AS PUTATIVE TARGETS FOR CHRONIC LOW BACK PAIN ALLEVIATION**

**Authors:**

Fozzato Stefania, Baranzini Nicolò, Bossi Elena**, Cinquetti Raffaella, Grimaldi Annalisa, Campomenosi Paola*, Surace Michele Francesco*.

*These authors share last authorship

** Corresponding Author

**Affiliations**:

**Fozzato Stefania,** M.D., Department of Medicine and Surgery, University of Insubria, Varese, Italy; [s.fozzato@uninsubria.it](mailto:s.fozzato@uninsubria.it) ; ORCID: 0000-0002-6368-2614;

**Baranzini Nicolò,** Ph.D., Department of Biotechnology and Life Sciences, University of Insubria, Varese, Italy; [n.baranzini@uninsubria.it](mailto:n.baranzini@uninsubria.it); ORCID:0000-0001-6996-4797;

**Bossi Elena,** Ph.D., Prof., Center for Neuroscience Research, University of Insubria, Interdisciplinary Research Centre for Pathology and Surgery of the Musculoskeletal System; Department of Biotechnology and Life Sciences, University of Insubria, Varese, Italy; [elena.bossi@uninsubria.it](mailto:elena.bossi@uninsubria.it) ; ORCID: 0000-0002-9549-2153;

**Cinquetti Raffaella,** Ph.D., Department of Biotechnology and Life Sciences, University of Insubria, Varese, Italy; [raffaella.cinquetti@uninsubria.it](mailto:raffaella.cinquetti@uninsubria.it); ORCID: 0000-0002-0000-8294;

**Grimaldi Annalisa**, Ph.D., Prof., Department of Biotechnology and Life Sciences, University of Insubria, Varese, Italy; [annalisa.grimaldi@uninsubria.it](mailto:annalisa.grimaldi@uninsubria.it); ORCID: 0000-0002-9258-7595;

**Campomenosi Paola**, Ph.D., Department of Biotechnology and Life Sciences, University of Insubria, Varese, Italy; [paola.campomenosi@uninsubria.it](mailto:paola.campomenosi@uninsubria.it); ORCID: 0000-0002-8853-1134;

**Surace Michele Francesco**, M.D., Prof., Interdisciplinary Research Centre for Pathology and Surgery of the Musculoskeletal System; Department of Biotechnology and Life Sciences, University of Insubria, Varese, Italy; [michele.surace@uninsubria.it](mailto:michele.surace@uninsubria.it); ORCID: 0000-0002-4645-8418;

**Corresponding author:**

**Bossi Elena:** Address: Via Dunant 3, Varese, 21100, VA, Italy

Phone number: +39 0332 421318

Email: [elena.bossi@uninsubria.it](mailto:elena.bossi@uninsubria.it);

**Supplementary file 1. Anamnestic and clinical data of patients included in the study**

**Patient 1:**

A 22-year-old man suffering from CLBP radiated in the posterior side of right thigh for 3 years. Instrumental investigations showed intraforaminal and paramedian hernia at L5-S1 level. He had been previously treated with corticosteroid therapies without benefits. In May 2016 herniectomy and discectomy of L5-S1 disc, associated to right selective hemilaminectomy were performed with improvement of symptoms. Specimens were obtained from the affected L5-S1 right joint, whereas a sample from the unaffected right L4-L5 level joint was used as control tissue. Samples obtained from this patient were insufficient for a complete analysis, thus expression at transcript level was skipped.

**Patient 2:**

A 33-year-old woman suffering from degenerative intervertebral disc disease at L4-L5 level. The patient was previously surgically treated to remove herniated left paramedian disc at the same level. In February 2016 she was surgically treated with posterolateral intervertebral fusion with pedicular screws and rods. During this surgery, samples from the articular capsule, bone and muscle of L4-L5 inter-apophyseal joints bilaterally were obtained. During pedicular screws insertion procedure, tissue from unaffected, pain-free L3-L4 level joint was collected and used as control.

**Patient 3:**

63-year-old man with lower back pain radiated in the posterior side of thighs, with worse symptoms on the right side, associated with intermittent neurological claudication. The patients were already treated with L4-L5 laminectomy in 2000. The instrumental investigations showed a spinal stenosis associated with discs protrusions in the L3-L5 levels. In October 2016 the patient underwent L3-S1 decompression surgery and stabilization with pedicular screws and rods associated with autologous postero-lateral bone grafting. Specimens were obtained from the affected L4-L5 and L5-S1 bilateral joints, whereas samples from L3-L4 level joints were collected as control tissue. Samples obtained from this patient were insufficient for a complete analysis.

**Patient 4:**

60-year-old man affected by lumbar spinal stenosis in the L4-L5 tract, with radicular compression of both the femoral and sciatic nerves associated with intermittent neurological claudication. The symptoms were worse on the right side. Instrumental investigations showed spondyloarthritis, L4-L5 anterior spondylolisthesis associated with multiple paramedian and intraforaminal hernias in the L3-L5 tract. In October 2016, a bilateral L4-L5 decompression and foraminotomy were performed, associated to posterolateral intervertebral fusion with pedicular screws and rods associated to autologous bone grafting. Specimens were obtained from the affected L4-L5 and L5-S1 bilateral joints, samples from L3-L4 level joints were used as control.

**Patient 5:**

54-year-old woman with L4-S1 lumbar spinal stenosis with bilateral radicular compression of sciatic nerve. The symptoms were worse on the right side. In December 2016 the patient was treated with L4-S1 right-selective hemilaminectomy and flavectomy, L5-S1 foraminotomy and posterolateral intervertebral fusion with pedicular screws and rods associated to autologous bone grafting on L4-S1 intervertebral spaces. Specimens were obtained from the affected L4-L5 and L5-S1 bilateral joints, whereas samples from L3-L4 level joints were used as control tissue.

**Patient 6:**

41-year-old woman with lumbar spinal stenosis L4-S1 with radicular deficit on right sciatic nerve. In 2007 L4-L5 and L5-S1 herniectomy, implantation of interspinous device (DIAM) at the L4-L5 level and left L5-S1 decompression were realized. However, due to persistent symptoms, in January 2017 was subjected to hemilaminectomy, flavectomy, L4-S1 right foraminotomy, removal of interspinous device, and L4-S1 posterolateral intervertebral fusion with pedicular screws and rods associated to autologous bone grafting. Specimens were obtained from the affected L4-L5 and L5-S1 bilateral joints, whereas samples from L3-L4 level joints were used as control tissue.

**Supplementary Table 1.**

| **TRPs** |  | **Immunogen peptide** | **Corresponding amino acid residues** |
| --- | --- | --- | --- |
| **TRPA1** | AB_2040232 | (C)NSTGIINETSDHSE | 747-760 of human TRPA1 (1st extracellular loop); |
| **TRPV1** | AB_2313819 | (C)EDAEVFKDSMVPGEK | 824-838 of rat TRPV1 (Intracellular, C-terminus region) |
| **TRPV2** | AB_2040266 | (C)KKNPTSKPGKNSASEE | 735-750 of rat TRPV2 (Intracellular, C-terminus region) |
| **TRPV4** | AB_2040264 | CDGHQQGYAPKWRAEDAPL | 853-871 of rat TRPV4 (Intracellular, C-terminus region) |
| **TRPM8** | AB_2040254 | SDVDGTTYDFAHC | 917-929 of human TRPM8 (3^rd^extracellular loop) |

**Supplementary Table 1.** Primary antibodies for immunofluorescence analysis, (Alomone labs, Jerusalem, Israel) all diluted 1:200 in blocking solution.

[**Supplementary Table**](https://www.ncbi.nlm.nih.gov/pmc/articles/PMC5222518/#pone.0169481.s001) **2.**

| **Gene** | **Accession no.** |  | **Sequence (5' 🡪 3')** | **Product**  **length (bp)** |
| --- | --- | --- | --- | --- |
| ***TRPA1*** | NM_007332.3 | **Fw** | GACCATGCTTCACAGAGCTTC | 108 |
|  |  | **Rv** | AGTGGAGAGCGTCCTTCAGA |  |
| ***TRPV1*** | NM_080705.4 | **Fw** | TGAGAGACCTGTGCCGTTTC | 106 |
|  |  | **Rv** | GACGGCAGGGAGTCATTCTT |  |
| ***TRPV2*** | NM_016113.5 | **Fw** | ATGCTGACCGTTGGCACTAA | 80 |
|  |  | **Rv** | GAAGCCCAGTTCACCTCCTC |  |
| ***TRPV4*** | NM_021625.5 | **Fw** | TCATGATCGGCTACGCTTCA | 80 |
|  |  | **Rv** | GGTCTGGTCCTCATTGCACA |  |
| ***TRPM8*** | NM_024080.5 | **Fw** | CAAAAGCCAACGACACCTCAG | 94 |
|  |  | **Rv** | GCAATCTCTTTCAGAAGACCCTT |  |
| ***B2M*** | NM_004048.4 | **Fw** | AGGCTATCCAGCGTACTCCA | 102 |
|  |  | **Rv** | ATGGATGAAACCCAGACACA |  |
| ***HPRT1*** | NM_000194.3 | **Fw** | TGCTGAGGATTTGGAAAGGGT | 95 |
|  |  | **Rv** | GGCCTCCCATCTCCTTCATC |  |
| ***GAPDH*** | NM_002046.7 | **Fw** | GAAGGTGAAGGTCGGAGTC | 172 |
|  |  | **Rv** | GAAGATGGTGATGGGATTTC |  |

[**Supplementary Table**](https://www.ncbi.nlm.nih.gov/pmc/articles/PMC5222518/#pone.0169481.s001) **2.** Sequences of primers used for gene expression analyses in this study.

**Supplementary Figure 1.**

**
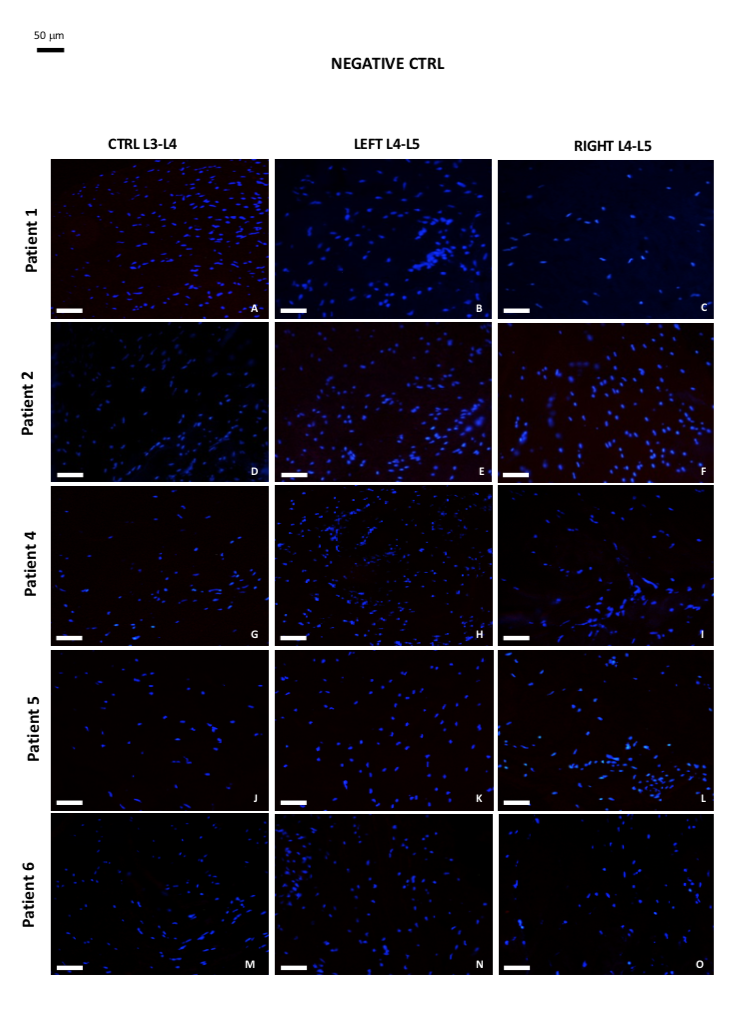
**

**Supplementary Figure 1.** Negative control of immunofluorescence experiments. Sections from control and pathological tissues, retrieved from patient 1 (A-C), patient 2 (D-F), patient 4 (G-I), patient 5 (J-L), patient 6 (M-O) were incubated only with the secondary antibodies, while primary antibodies against TRPA1, TRPV1, TRPV2, TRPV4, TRPM8 were omitted. No red signal is detectable in any of the examined tissues. Nuclei counterstained with DAPI (blue). Bars: 50 µm.

**Supplementary Figure 2.**


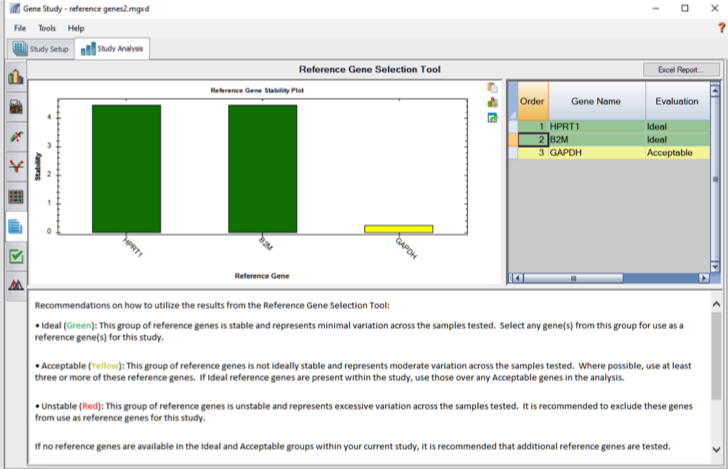


**Supplementary Figure 2.** Screenshot of the “Reference Gene Selection Tool” from the CFX Maestro software, based on GeNorm, showing the stability of the three reference genes initially tested in this study. Green colored bars indicate ideal reference genes (stable and showing minimal variation across samples); yellow colored bars indicate acceptable reference genes (not ideally stable and showing moderate variation across samples).

**Supplementary Figure 3.**

**Supplementary Figure 3.** Calibration curves for the assays used in this study. All efficiencies (E) were comprised between 95 and 105% and *B2M,* among the two reference genes analyzed, showed the efficiency most similar to those of the genes under investigation.

**Supplementary Figure 4.**

**Supplementary Figure 4.** Melting curves of amplicons obtained with the different assays used in this study. The graphs show a single peak, indicating that a single amplification product is obtained.
